# Efecto de la exposición previa a COVID-19, ocurrencia de brotes y tipo de vacuna en la respuesta inmune humoral de adultos mayores institucionalizados

**DOI:** 10.1590/0102-311XES155023

**Published:** 2024-10-11

**Authors:** Fernanda Aguirre, María Jimena Marro, Pamela E. Rodriguez, Pablo Rall, Esteban A. Miglietta, Lucía A. López Miranda, Verónica Poncet, Carla A. Pascuale, Christian A. Ballejo, Tamara Ricardo, Yanina Miragaya, Andrea Gamarnik, Andrés H. Rossi, Andrea P. Silva

**Affiliations:** 1 Instituto Nacional de Epidemiología “Dr. Juan H. Jara”, Administración Nacional de Laboratorios e Institutos de Salud “Dr. Carlos G. Malbrán”, Mar del Plata, Argentina.; 2 Fundación Instituto Leloir, Buenos Aires, Argentina.; 3 Consejo Nacional de Investigaciones Científicas y Técnicas, Buenos Aires, Argentina.; 4 Instituto Nacional de Servicios Sociales para Jubilados y Pensionados, Buenos Aires, Argentina.

**Keywords:** Infecciones por Coronavirus, Inmunogenicidad Vacunal, Salud del Anciano Institucionalizado, Pandemias, Coronavirus Infections, Vaccine Immunogenicity, Health of Institutionalized Elderly, Pandemics, Infecções por Coronavírus, Imunogenicidade da Vacina, Saúde do Idoso Institucionalizado, Pandemias

## Abstract

El objetivo de este trabajo fue evaluar los factores explicativos de la respuesta inmune humoral en adultos mayores de establecimientos de estancia prolongada de Buenos Aires, Argentina, hasta 180 días post vacunación. Se utilizó un diseño de cohorte abierta, prospectiva, multicéntrica, con voluntarios que recibieron dos dosis de vacunas Sputnik V, Sinopharm o AZD1222. Se analizaron muestras de plasma en los tiempos 0, 21 días post primera dosis, 21 días post segunda dosis, 120 y 180 días post primera dosis. Se ajustaron modelos lineales marginales y aditivos generalizados mixtos para evaluar el comportamiento de la concentración de anticuerpos IgG anti-Spike en el tiempo según grupo de exposición (*naïve*/no-*naïve*) y vacuna. Las covariables analizadas fueron: ocurrencia de brote de COVID-19 en establecimientos de estancia prolongada y comorbilidades. Se incluyeron en el análisis 773 participantes con una mediana de edad de 83 años (RIQ: 76-89). Al final del estudio, los niveles de anticuerpos del grupo *naïve*: Sinopharm fueron significativamente menores que el resto de los grupos (p < 0,05); los del no-*naïve*: Sinopharm resultaron similares a los *naïve* que recibieron AZD1222 (p = 0,945) o Sputnik V (p = 1). Los participantes expuestos a brotes en establecimientos de estancia prolongada presentaron niveles de anticuerpos significativamente mayores, independientemente del grupo de exposición y la vacuna (p < 0,001). Concluimos que la exposición previa a COVID-19, el tipo de vacuna y la pertenencia a un establecimiento de estancia prolongada con antecedente de brote son factores a considerar frente a futuros eventos epidémicos con dinámicas de transmisión y mecanismos inmunológicos similares al COVID-19, en poblaciones similares a la analizada en este trabajo.

## Abstract (en)

This study evaluated the explanatory factors of humoral immune response in older adults admitted to long-term care institutions in Buenos Aires, Argentina, up to 180 days after vaccination. An open-label, prospective, multicenter cohort study was conducted with volunteers who received two doses of the Sputnik V, Sinopharm, or AZD1222 vaccines. Plasma samples were analyzed at 0 and 21 days after the first dose, 21 days after the second dose, and 120 and 180 days after the first dose. Marginal linear models and generalized additives mixed models were adjusted to determine the behavior of anti-spike IgG antibody concentration over time according to exposure group (naïve/no-naïve) and vaccine. Occurrence of an outbreak of COVID-19 in long-term care institutions and comorbidities were the covariates analyzed. A total of 773 participants were included, with a mean age of 83 years (IQR: 76-89). Results showed that antibody levels in the naïve: Sinopharm group were significantly lower to the other groups (p < 0.05). Antibody levels in the no-naïve: Sinopharm group were similar to those in the naïve group who received AZD1222 (p = 0.945) or Sputnik V (p = 1). Participants exposed to outbreaks in long-term care institutions had significantly higher antibody levels, regardless of exposure group and vaccine (p < 0.001). In conclusion, previous exposure to COVID-19, type of vaccine, and admittance into a long-term care institution with a history of outbreaks are factors to be considered in future epidemic events with transmission dynamics and immunological mechanisms similar to COVID-19, in populations similar to the one analyzed.

## Resumo

Este estudo teve como objetivo avaliar os fatores explicativos da resposta imune humoral em idosos em instituições de longa permanência em Buenos Aires, Argentina, até 180 dias após a vacinação. Foi realizado um estudo de coorte aberto, prospectivo e multicêntrico, com voluntários que receberam duas doses das vacinas Sputnik V, Sinopharm ou AZD1222. As amostras de plasma foram analisadas nos tempos 0, 21 dias após a primeira dose, 21 dias após a segunda dose, 120 e 180 dias após a primeira dose. Os modelos lineares marginais e os aditivos generalizados mistos foram ajustados para determinar o comportamento da concentração de anticorpos IgG anti-Spike ao longo do tempo de acordo com o grupo de exposição (*naïve*/*no-naïve*) e vacina. As covariáveis analisadas foram ocorrência de pico de COVID-19 nas instituições de longa permanência e comorbidades. Foram incluídos 773 participantes, com média de idade de 83 anos (IIQ: 76-89). Os resultados apontaram níveis de anticorpos do grupo *naïve*: Sinopharm significativamente mais baixos do que os outros grupos (p < 0,05); e as variáveis do grupo *no-naïve*: Sinopharm foram semelhantes à do grupo *naïve* que recebeu AZD1222 (p = 0,945) ou Sputnik V (p = 1). Os participantes expostos a picos nas instituições de longa permanência apresentaram níveis de anticorpos significativamente maiores, independentemente do grupo de exposição e da vacina (p < 0,001). Conclui-se que a exposição prévia à COVID-19, tipo de vacina e adesão a uma instituição de longa permanência com histórico de pico são fatores a serem considerados em futuros eventos epidêmicos com dinâmica de transmissão e mecanismos imunológicos semelhantes à COVID-19, em populações semelhantes à analisada neste trabalho.

## Introducción

Desde la declaración de la pandemia por parte de la Organización Mundial de la Salud (OMS) en marzo de 2020, la enfermedad por coronavirus 2019 (COVID-19) ha producido un impacto inconmensurable en términos sociales, económicos y sanitarios a escala global. Entre los grupos poblacionales más vulnerables se ubicaron desde el inicio los adultos mayores. Los indicadores de morbimortalidad reflejaban riesgo aumentado de hospitalización y muerte por COVID-19 en este grupo etario ^1^ ^,^ ^2^; el envejecimiento constituía una preocupación específica que dio lugar al surgimiento de nuevos interrogantes sobre el desempeño de la respuesta inmune a la vacuna contra COVID-19 en este subgrupo ^3^ ^,^ ^4^.

A su vez, los establecimientos de estancia prolongada de adultos mayores constituyeron tempranamente un blanco de atención en todo el mundo a raíz de la ocurrencia de brotes de COVID-19 ^5^ ^,^ ^6^ ^,^ ^7^. La condición de fragilidad de sus residentes ^8^ sumada a la permanencia en lugares semicerrados potenciaba el riesgo de contagios y alertaba sobre la saturación de los servicios de salud.

Durante los primeros meses de la epidemia en Argentina, las estrategias para afrontar la situación se centraron en el aislamiento social preventivo y obligatorio que exceptuaba a las personas afectadas a las actividades esenciales, el refuerzo de la infraestructura sanitaria, la vigilancia epidemiológica y el control de brotes ^9^ ^,^ ^10^. Una vez aprobadas las primeras vacunas contra COVID-19, se convirtieron en una herramienta de prevención primaria fundamental en la reducción del riesgo de morbimortalidad asociada a la enfermedad. Argentina, siguiendo las recomendaciones internacionales, inició en diciembre de 2020 un plan de vacunación masiva que consideraba a los adultos mayores en segundo orden de prioridad, luego del personal de salud ^11^. Las vacunas aplicadas fueron rAd26-rAd5 (Sputnik V -Instituto Gamaleya, Rusia), ChAdOx1 (AZD1222 -AstraZeneca/Oxford, Reino Unido), y BBIBP-CorV (Sinopharm -Instituto de Beijing de Productos Biológicos, China), que se mostraron eficaces para inhibir la infección viral ^12^ ^,^ ^13^ ^,^ ^14^. En particular, la estrategia de vacunación en la provincia de Buenos Aires fue implementada en los establecimientos de estancia prolongada por personal del Programa de Asistencia Médica Integral (PAMI), dependiente del Instituto Nacional de Servicios Sociales para Jubilados y Pensionados.

Dada la escasez de estudios sobre el efecto de la vacunación con estas plataformas en adultos mayores en la región, el objetivo de este trabajo fue evaluar los factores explicativos de la respuesta inmune humoral en el tiempo en adultos mayores de establecimientos de estancia prolongada de la provincia de Buenos Aires, a los 180 días post vacunación.

## Métodos

Se utilizó un diseño de cohorte abierta, prospectiva, multicéntrica. Se invitó a participar a residentes de establecimientos de estancia prolongada de las Unidades de Gestión Local (UGL) del PAMI número VII (La Plata y Berisso), X (Almirante Brown, Avellaneda, Esteban Echeverría, Ezeiza, Lanús y Lomas de Zamora) y XI (Mar del Plata-Batán) de la provincia de Buenos Aires, que no hubieran concretado la vacunación al momento de inicio del estudio. Dichas UGL cuentan con poblaciones de 816.419, 2.673.961 y 659.462 habitantes respectivamente, de los cuales se estima que el 16,40% son adultos mayores ^15^ ^,^ ^16^. Se incluyó a todos los residentes de dichos establecimientos de estancia prolongada que hubieran aceptado voluntariamente recibir la vacuna contra COVID-19 y firmaran el consentimiento informado para participar de la investigación; se excluyeron aquellos con contraindicaciones para la punción venosa. El período de reclutamiento de participantes abarcó del 19 marzo al 20 mayo del 2021.

En el marco del plan de vacunación, los participantes recibieron dos dosis de las vacunas con intervalos variables: Sputnik V (Mediana [Me]: 62, rango intercuartílico [RIQ]: 55-73 días), Sinopharm (Me: 32, RIQ: 30-50 días) o AZD1222 (Me: 49, RIQ: 45-52 días). Se obtuvieron muestras de plasma (EDTA) por punción venosa en tubos previamente rotulados con el código anonimizado asignado al participante (ID), en cada establecimientos de estancia prolongada incluido en el estudio. Los tiempos de toma de muestras fueron: día 0 (línea de base), 21 días después de la primera dosis, 21 días después de la segunda dosis, 120, 180 y 365 días después de la primera dosis. Previo a la obtención de las muestras, se acordaba con los responsables de los establecimientos de estancia prolongada la fecha y horario de concurrencia del equipo de campo al establecimiento, para minimizar las pérdidas de seguimiento.

La detección de anticuerpos de tipo IgG frente a SARS-CoV-2 se realizó por la técnica de ELISA, con el test COVID-AR IgG (COVID-AR IgG, CONICET Leloir-Lemos S.R.L. 2020) que determina la IgG específica del dominio de unión al receptor (RBD, por sus siglas en inglés) de la glicoproteína spike del SARS-CoV-2 como así también IgG específica anti-Spike ^17^. Las determinaciones serológicas se realizaron en el laboratorio del Instituto Nacional de Epidemiología “Dr. Juan H. Jara” (INE) y en la Fundación Instituto Leloir, bajo un nivel de bioseguridad II.

El personal médico de los establecimientos de estancia prolongada se encargó del registro de datos sobre comorbilidades de los participantes en la línea de base y ocurrencia de COVID-19 durante el período de seguimiento. El equipo de investigación registró los títulos obtenidos de las determinaciones de anticuerpos y la ocurrencia de brotes de COVID-19 en los establecimientos de estancia prolongada y defunciones de participantes durante el estudio. Los datos de ocurrencia de COVID-19 durante el seguimiento y los óbitos fueron validados con su notificación en el Sistema Nacional de Vigilancia de la Salud (SNVS 2.0).

Se excluyó del análisis estadístico a aquellos participantes que abandonaron voluntariamente el estudio o fallecieron antes de la primera instancia de seguimiento (día 21 después de primera dosis), así como los que tenían información incompleta sobre comorbilidades y exposición a brotes de COVID-19 en el establecimiento de estancia prolongada. También fueron excluidos los datos de anticuerpos IgG del último tiempo del seguimiento (365 días de la primera dosis), debido a que se introdujo una dosis de refuerzo en el marco del plan nacional de vacunación. Se consideró seroconversión: (a) en aquellos participantes cuyo nivel basal de anticuerpos fue no detectable, cualquier título detectable en la medición inmediata posterior a completar el esquema de dos dosis y (b) en quienes tuvieron valores detectables en la medición basal, un aumento de cuatro veces o más del título basal en la medición inmediata posterior a completar el esquema de dos dosis.

Para los diferentes modelos, se seleccionó como variable respuesta la concentración de anticuerpos IgG anti-Spike expresada en unidades arbitrarias (UA). Los valores por debajo del límite de detección para cada tiempo se imputaron mediante un método de regresión lineal para datos censurados hacia la izquierda, utilizando el paquete *censml* ^18^. Este método ajusta un modelo de regresión lineal, estimando los datos por debajo del límite de detección de la técnica a partir del valor o valores de *cut-off* proporcionados. El porcentaje de valores imputados fue 57,3%, 37,5%, 7,4%, 10,4% y 14,2% para día cero, 21 días después de la primera dosis, 21 días después de la segunda dosis, 120 y 180 días después de la primera dosis, respectivamente. A fin de controlar la presencia de valores extremos, se realizó la transformación logarítmica de la variable respuesta.

Se definieron dos grupos de exposición: *naïve* (sin antecedentes de COVID-19 y seronegativos en la línea de base) y no-*naïve* (con antecedente de COVID-19 o seropositivos en la línea de base). A fin de evitar el uso de interacciones triples, se definió la variable explicativa “tratamiento” compuesta por seis niveles: *naïve*: Sinopharm, *naïve*: Sputnik V, *naïve*: AZD1222, no-*naïve*: Sinopharm, no-*naïve*: Sputnik V, no-*naïve*: AZD1222. Se evaluó la presencia de autocorrelación temporal entre observaciones mediante gráficos de autocorrelación (ACF) y test de Ljung-Box.

Se valoró el efecto de la interacción entre el tratamiento (grupo de exposición: vacuna) y el tiempo categórico (0, 21, 42, 120, 180 días) mediante modelos lineales marginales por el método de mínimos cuadrados generalizados (GLS, por sus siglas en inglés) ^19^; la presencia de medidas repetidas se controló incorporando un término autorregresivo continuo de orden 1 (corCAR1) que controla por ID y establecimientos de estancia prolongada. Se evaluaron como posibles predictores: sexo (mujer, varón), grupo etario (60-83 años, > 83 años), COVID-19 durante el estudio (sí, no), ocurrencia de brotes de COVID-19 en el establecimiento de estancia prolongada (sí, no), diabetes mellitus (sí, no), hipertensión arterial (sí, no), insuficiencia cardíaca (sí, no) e inmunodeficiencia (sí, no). Se excluyeron del análisis las comorbilidades obesidad severa (sí, no), enfermedad pulmonar obstructiva crónica (EPOC) (sí, no) y enfermedad renal crónica (sí, no) debido a la baja frecuencia que presentaron en la cohorte. Se ajustaron dos modelos saturados considerando: (i) homogeneidad y (ii) heterogeneidad de varianzas (estructura varIdent). Ambos modelos se compararon en base al criterio de información de Akaike (AIC, por sus siglas en inglés). Al modelo más parsimonioso, se le realizó un procedimiento *backward* manual de selección de variables. En relación con el mismo, se describió el comportamiento no lineal de los anticuerpos en el tiempo, utilizando modelos aditivos generalizados mixtos (GAMM, por sus siglas en inglés). Se incluyó una curva de suavizado para la interacción entre el tratamiento (grupo: vacuna) y el tiempo (días), ajustada por exposición a brotes de COVID-19 en el establecimiento de estancia prolongada. Se utilizaron funciones de suavizado con *splines* cúbicas con cinco nodos y se controlaron las medidas repetidas, incluyendo efectos aleatorios del participante y del establecimiento de estancia prolongada de procedencia. Las inferencias se realizaron en base a la parte paramétrica del GAMM.

El análisis estadístico se realizó en software R versión 4.3.1 (http://www.r-project.org), utilizando los paquetes *nlme* ^20^ y *mgcv* ^21^. Los *scripts* de R utilizados para el análisis de datos se encuentran disponibles en: https://zenodo.org/records/13741499.

Con relación a las consideraciones éticas, se tomaron todas las precauciones para garantizar la confidencialidad de la información. Previo a la realización de cualquier procedimiento relacionado con el estudio, se obtuvo el consentimiento informado de los participantes. El protocolo de investigación fue aprobado por el Comité de Ética de Investigación del Hospital Bernardo Houssay de Mar del Plata, el día 8 de marzo de 2021 (código: 03/2021).

## Resultados

Se colectaron muestras de sangre de 850 participantes, de los cuales 34 (4%) no completaron la primera instancia de seguimiento (23 fallecidos, 11 abandonaron voluntariamente el estudio) y 43 (5,1%) tenían datos incompletos respecto a comorbilidades o exposición a brotes de COVID-19 en el establecimientos de estancia prolongada.

Se incluyó en el análisis un total de 773 participantes que residían en 56 establecimientos, 27 (48,2%) de la UGL XI, 18 (32,1%) de la X y 11 (19,6%) de la VII. No se detectaron diferencias significativas respecto a la distribución por sexo (p = 1,00) o edad (p = 0,729) entre los pacientes incluidos en el análisis (n = 773) y aquellos que se excluyeron (n = 77). El período de seguimiento fue de 180 días; 442 participantes (57,2%) pertenecían al grupo *naïve* y 331 (42,8%) al no-*naïve*. El 57% de los participantes del grupo *naïve* (252) y 69,5% del no-*naïve* (230) recibieron la vacuna Sputnik V, 23,1% del grupo *naïve* (102) y 16% del no-*naïve* (53) Sinopharm; 19,9% del grupo *naïve* (88) y 14,5% del no-*naïve* (48) recibieron AZD1222. La edad de los participantes tuvo un mínimo de 60 y un máximo de 103 años (Me: 83, RIQ: 76-89 años). Las mujeres tuvieron una mediana de edad de 86 años (RIQ: 78-91 años), que resultó significativamente mayor (p < 0,001) que la observada en hombres (Me: 76, RIQ: 71-83 años).

Al considerar la extracción inmediatamente posterior a la segunda dosis (Me: 21, RIQ: 21-22 días), se observó seroconversión en 607 participantes (84,74%) sobre 725 que contaron con datos para establecer la seroconversión en ese momento. Se observaron diferencias estadísticamente significativas respecto al grupo que no tuvo seroconversión en edad (p = 0,0018) y tipo de vacuna (p < 0,001); no se encontraron diferencias para sexo. La mediana de edad de quienes seroconvirtieron fue de 81,92 años (RIQ: 75-89), en tanto que la de quienes no, fue de 84,62 (RIQ: 78-91). La mayor proporción entre quienes no seroconvirtieron correspondió a individuos vacunados con Sinopharm (57,63%), en tanto que la mayor proporción entre quienes seroconvirtieron se observó en individuos vacunados con Sputnik V (68,37%).

En una mediana de 67 días (RIQ: 57-75) medidos desde la segunda dosis, 371 participantes (61,12%) de aquellos que habían seroconvertido, experimentaron descenso en sus niveles de anticuerpos. Si se consideran los 773 participantes, 44 (5,69%) no elevaron su nivel basal de anticuerpos en las mediciones efectuadas durante 6 meses de seguimiento, de los cuales 33 (4,26%) mantuvieron un nivel no detectable durante la totalidad de ese período.

**Figura 1:**
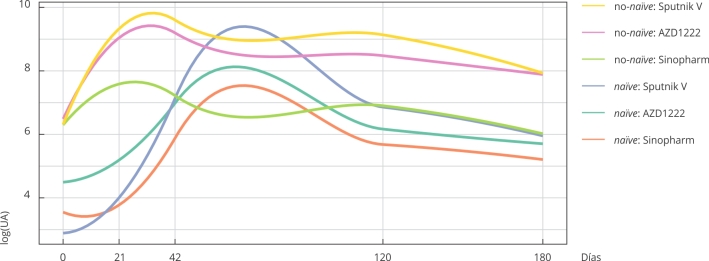
Curvas ajustadas de los perfiles promedio del nivel de anticuerpos en el tiempo según interacción entre grupo de exposición y vacuna recibida.

Se observaron distintos comportamientos de la curva de anticuerpos para cada combinación de grupo y vacuna. La Figura 1 muestra los datos representados mediante curvas de suavizado obtenidas a partir del método de regresión local (*loess*). El test de Ljung-Box mostró que la autocorrelación temporal fue estadísticamente significativa (p < 0,001).

log(UA): logaritmo de la concentración de anticuerpos.

Se ajustaron dos GLS saturados candidatos, utilizando como variables explicativas tratamiento, tiempo categórico (y su interacción), sexo, grupo etario, brote de COVID-19 en el establecimiento de estancia prolongada, infección con COVID-19 durante el estudio, diabetes mellitus, hipertensión arterial, insuficiencia cardíaca e inmunodeficiencia, controlando medidas repetidas mediante una estructura de autocorrelación temporal corCAR1 y evaluando (i) homogeneidad o (ii) heterogeneidad de varianzas. De ellos, el modelo más parsimonioso fue el que consideraba varianzas heterogéneas (ΔAIC: 112,7). A partir del mismo, se obtuvo un modelo final que tenía como variables explicativas al tratamiento, el tiempo categórico, la interacción entre ambas y la exposición a brotes de COVID-19 en el establecimientos de estancia prolongada (Tabla 1).

**Tabla 1:** Selección de variables explicativas en el modelo de mínimos cuadrados generalizados (GLS, por sus siglas en inglés) con estructura de correlación temporal autorregresiva continua de primer orden (corCAR1) y heterogeneidad de varianzas (varIdent).

Modelo	Brote en el establecimiento de estancia prolongada	COVID-19	Sexo	Grupo etario	Diabetes mellitus	Hipertensión arterial	Insuficiencia cardíaca	Inmunodeficiencia	AIC	Delta
GLS2g	+	-	-	-	-	-	-	-	13.789,08	0,00
GLS2f	+	-	-	-	+	-	-	-	13.790,45	1,37
GLS2e	+	-	-	-	+	+	-	-	13.793,59	4,50
GLS2d	+	-	-	-	+	+	+	-	13.795,80	6,72
GLS2c	+	-	-	-	+	+	+	+	13.796,92	7,83
GLS2b	+	-	-	+	+	+	+	+	13.801,40	12,32
GLS2a	+	-	+	+	+	+	+	+	13.806,04	16,96
GLS2	+	+	+	+	+	+	+	+	13.809,63	20,55

AIC: criterio de información de Akaike.

Nota: todos los modelos incluyen la interacción entre tiempo y tratamiento y efecto aleatorio para paciente y hogar de procedencia. Los signos + y - indican inclusión o exclusión de la variable explicativa.

**Figura 2:**
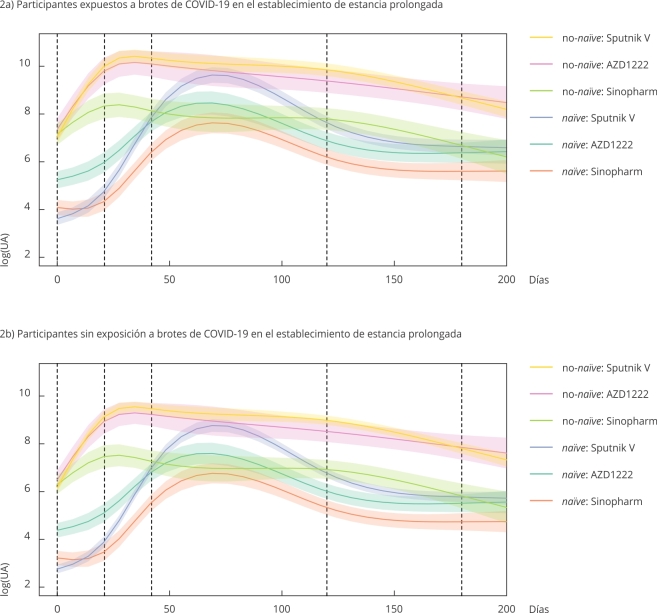
Funciones de suavizado del modelo aditivo generalizado mixto (GAMM, por sus siglas en inglés) con término autorregresivo corCAR1 y heterogeneidad de varianzas para el comportamiento de los anticuerpos en el tiempo, con efectos aleatorios para paciente y hogar de procedencia.

**Figura 3:**
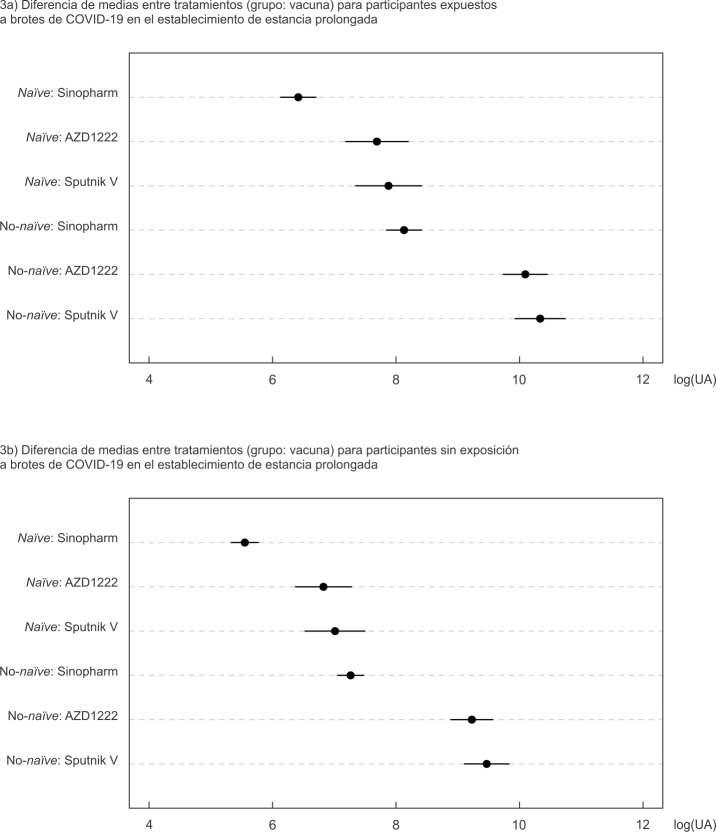
Gráfico de los componentes paramétricos del modelo aditivo generalizado mixto (GAMM, por sus siglas inglés) con término autorregresivo corCAR1 y heterogeneidad de varianzas, ajustado por exposición a brotes de COVID-19 en el establecimiento de estancia prolongada, con efectos aleatorios para paciente y hogar.

Tomando como base el GLS final, se modeló la respuesta no lineal utilizando un modelo GAMM con estructuras corCAR1, varIdent y efectos aleatorios del participante y el establecimiento de estancia prolongada. Este modelo pudo explicar el 61,5% de la variabilidad observada. Los grados de libertad efectivos para las curvas de suavizado de cada nivel de la variable tratamiento oscilaron entre 3,54 y 3,98, sugiriendo un comportamiento fuertemente no lineal (Figura 2). La parte paramétrica del modelo presentó un comportamiento similar al observado para el GLS, en el que el grupo *naïve*: Sinopharm presentó niveles de anticuerpos significativamente menores que el resto de las combinaciones de grupo de exposición y vacuna, mientras que el no-*naïve*: Sinopharm mostró niveles de anticuerpos similares a los registrados para los *naïve* que recibieron AZD1222 o Sputnik V. También puede observarse que los participantes expuestos a brotes de COVID-19 en el establecimiento de estancia prolongada presentaron niveles de anticuerpos mayores que aquellos que no, independientemente de si eran parte del grupo *naïve* o no-*naïve* y de la vacuna que recibieron (Figura 3).

log(UA): logaritmo de la concentración de anticuerpos.

log(UA): logaritmo de la concentración de anticuerpos.

## Discusión

En este trabajo se encontró que la exposición previa a COVID-19, el tipo de vacuna recibida y la pertenencia a un establecimiento de estancia prolongada en el que ocurrió un brote de COVID-19 durante el período de seguimiento de los voluntarios, fueron factores asociados a la respuesta inmune humoral. Por otra parte, los efectos de la edad, el antecedente de insuficiencia cardíaca o inmunodeficiencia se diluyeron con la aplicación del análisis multivariado. Teniendo en cuenta esto, nuestra investigación agrega valor sobre la contribución de diferentes factores sobre la respuesta inmune humoral frente a tres plataformas de vacunas (Sputnik V, AZD1222 y Sinopharm) aplicadas en Argentina en adultos mayores institucionalizados.

En una publicación previa ^22^, demostramos que las tres plataformas vacunales mencionadas fueron efectivas en el desarrollo de la respuesta inmune humoral hasta los 6 meses de iniciado el esquema de vacunación en esta población, medida como título de anticuerpos anti-Spike y la capacidad neutralizante. Un estudio, que evaluó la efectividad de estas mismas vacunas en la reducción del riesgo de infección y muerte en adultos mayores de la población general de Argentina, determinó que las personas con edad mayor o igual a 80 años exhibieron una menor efectividad en la prevención de la muerte por COVID-19 respecto de los más jóvenes luego de la primera dosis para las tres vacunas, que dejó de observarse con la segunda dosis de Sputnik V y AZD1222 ^23^. Otros trabajos de Argentina sumaron evidencia sobre la efectividad de estas vacunas en la reducción del riesgo de diversos desenlaces asociados a COVID-19 en adultos mayores ^24^ ^,^ ^25^. Nuestra investigación, por su parte, permitió demostrar, a partir de la interacción entre la exposición previa a COVID-19 y el tipo de vacuna, que los participantes no-*naïve* que recibieron Sputnik V o AZD1222 fueron los que exhibieron una respuesta inmune de mayor cuantía y sostenida hasta los 180 días posteriores a la vacunación.

En el caso de BBIBP-CorV, nuestros resultados indican títulos menores de anticuerpos, tanto para pacientes *naïve* como no-*naïve*. Esto es esperable, dado que al ser una vacuna a virus inactivado es menos inmunogénica que las restantes analizadas en este estudio, que son vacunas con vectores adenovirales con replicación defectuosa. Las vacunas a vectores adenovirales tienen la capacidad de replicarse en las células del huésped, lo que significa que pueden generar múltiples copias del antígeno objetivo y estimular una respuesta inmunitaria más potente. En contraste, los virus inactivados no pueden replicarse, por lo que presentan una cantidad limitada de antígeno a las células del sistema inmune ^26^ ^,^ ^27^. Asimismo, los resultados concuerdan con lo indicado en el informe sobre esta vacuna del grupo de expertos de la OMS, que evidenció alta seropositividad en adultos mayores, pero títulos más bajos en comparación con los adultos más jóvenes, tanto para anticuerpos de unión como para anticuerpos neutralizantes ^28^.

Si bien varias investigaciones observaron una respuesta inmune a las vacunas contra COVID-19 de menor magnitud en las personas de edad avanzada ^29^ ^,^ ^30^ ^,^ ^31^, en nuestro trabajo el efecto de la edad se diluyó en el análisis multivariado, resultando otros factores con mayor fuerza explicativa. Además, hay que recordar que la población de este estudio es una población envejecida (Me: 83 años), en la que existe un deterioro del sistema inmune producto del envejecimiento similar para todos los participantes, por lo que es esperable no encontrar diferencias al respecto. En este mismo sentido, una revisión sistemática y metaanálisis no demostró diferencias significativas en el efecto inmune de diferentes vacunas contra COVID-19 en adultos mayores respecto de los jóvenes ^32^.

Estudios llevados a cabo en población similar, tales como GeroCovid Vax, encontraron que residentes con diabetes tipo 2 presentaban una respuesta de anticuerpos más débil a la vacunación. En nuestra cohorte, los modelos no dieron cuenta de tal asociación. Esta diferencia podría ser explicada porque en nuestro caso todos los residentes evaluados recibieron dos dosis de vacunas, en tanto que en el citado estudio aproximadamente el 58% de los participantes contaban con la segunda dosis y además por las diferentes vacunas aplicadas ^33^.

El análisis estratificado por exposición previa a COVID-19 nos permitió demostrar una respuesta de anticuerpos más precoz, elevada y sostenida en el grupo con antecedentes de exposición previa a COVID-19. Estos hallazgos son consistentes con otros trabajos que evaluaron diferentes plataformas de vacunas contra COVID-19 ^31^ ^,^ ^34^ ^,^ ^35^ ^,^ ^36^ ^,^ ^37^ indicando el rol de la inmunidad híbrida ^38^. Diversos estudios sugieren que la vacunación después de la infección natural incrementa los niveles de anticuerpos neutralizantes contra la proteína Spike y las células T específicas ^39^. La infección pulmonar por el SARS-CoV-2 y la inmunización, ya sea con vacunas a virus inactivado o con vectores virales, implican distintos entornos inflamatorios para la presentación inmune de la proteína Spike. La infección por SARS-CoV-2 recluta células T efectoras en el tejido pulmonar, generando células T y B de memoria residentes en el tejido ^40^. Las células T, que ingresan al tejido pulmonar o interactúan con células de la inmunidad innata en los ganglios linfáticos pulmonares, pueden encontrar un entorno inflamatorio distinto del que está presente durante la inoculación de un antígeno por la vía intramuscular. La ventaja protectora de la inmunidad híbrida probablemente surja de la combinación de un mayor número de células B de memoria específicas para diferentes antígenos de SARS-CoV-2, mayores títulos de anticuerpos neutralizantes y el perfil de citoquinas de las células T CD4+ generado en la infección ^41^.

Por otro lado, el efecto *booster* provocado por la ocurrencia de brotes epidémicos de COVID-19 ha sido demostrado a partir del registro de seropositividad de anticuerpos en personas sin diagnóstico previo de la enfermedad ^42^ ^,^ ^43^. Si bien las características clínico-epidemiológicas de los brotes se modificaron a partir de la introducción de la vacunación en los establecimientos de estancia prolongada ^44^ ^,^ ^45^, estos eventos epidémicos siguieron aconteciendo, de allí la importancia de considerarlos en el análisis ajustado; el hecho de no hacerlo podría llevar a sobreestimación del efecto de la vacuna ^46^. En un contexto pre vacunación, encontramos un efecto protector de dicha exposición frente a nuevos brotes, medido como la presencia de anticuerpos anti-Spike en residentes de un establecimientos de estancia prolongada que había atravesado un brote unos pocos meses antes y no habían tenido confirmación diagnóstica de la infección ^47^. En esta investigación, el hecho de ser residente de un establecimientos de estancia prolongada donde ocurrió un brote de COVID-19 fue una variable relevante en la generación de la respuesta inmune.

Entre las fortalezas de este trabajo, subrayamos el aporte al conocimiento de los factores asociados a la dinámica de la respuesta inmune en una subpoblación vulnerable. Cabe resaltar la relevancia de esta contribución que se inscribe en la experiencia de una región particularmente afectada por la pandemia ^48^ en la que, a pesar de un consenso global sobre la elevada incidencia de brotes por SARS-CoV-2 en establecimientos de estancia prolongada ^49^, la información proveniente de los sistemas de vigilancia epidemiológica es escasa. A su vez, el trabajo aporta al conocimiento sobre diferentes plataformas de vacunas contra COVID-19 que no han sido las más frecuentemente evaluadas en la literatura internacional, a pesar de su amplio uso en varias regiones del mundo. Con relación a la validez interna del estudio, la técnica empleada en las determinaciones serológicas (*kit* COVIDAR) ha demostrado ser robusta en diversos estudios ^17^ ^,^ ^31^ ^,^ ^50^.

Como limitaciones, destacamos que no se pudo realizar el análisis a los 365 días después de la vacunación, tal como se estipuló inicialmente, debido a que las dosis de refuerzo adicionadas en el marco del plan nacional de vacunación modificaron las condiciones del contexto. Esto hubiese requerido la extracción de muestras de sangre adicionales en los participantes, lo cual fue inviable debido a las características de fragilidad de la población estudiada. Con la intención de minimizar el sesgo de selección resultante de las pérdidas del seguimiento, se sostuvieron estrategias de comunicación asiduas entre referentes del equipo de investigación y responsables de los establecimientos de estancia prolongada. Otra limitación fue la exclusión de registros con datos ausentes sobre comorbilidades que produjo una disminución del tamaño muestral. Si bien los participantes incluidos en el análisis tuvieron una distribución por sexo y edad similar a los que se excluyeron, el efecto observado respecto de las comorbilidades puede haber estado sesgado. Además, respecto al análisis estadístico, no se consideraron variables propias del establecimiento de estancia prolongada debido en parte a la dificultad de obtención de las mismas, por lo que no se pudo dar cuenta del efecto de ciertas características del contexto en la respuesta inmune humoral. Consideramos, sin embargo, que la variabilidad entre las particularidades del entorno de los establecimientos de estancia prolongada podría captarse principalmente mediante el resultado de la ocurrencia de brotes (variable incluida en la investigación) y de haber utilizado el establecimiento de estancia prolongada como efecto aleatorio en los modelos. Finalmente, si bien los modelos GAMM presentan términos paramétricos, se debe proceder con cuidado en la interpretación de los coeficientes y valores de p obtenidos a partir de los mismos, siendo preferible basarse en los resultados del GLS para la inferencia y del GAMM para describir la relación no lineal.

Como conclusión, en el contexto de incertidumbre en el que se desató la pandemia, valoramos relevante evaluar las variables involucradas en la obtención de la respuesta inmune a las vacunas contra COVID-19 en adultos mayores, para orientar decisiones en salud pública. En particular, consideramos que los factores explicativos que encontramos deberían ser tomados en cuenta frente a futuros eventos epidémicos con dinámicas de transmisión y mecanismos inmunológicos similares al COVID-19, en poblaciones similares a la analizada en este trabajo.
